# Awake craniotomy in brain tumors - Technique systematization and the state of the art

**DOI:** 10.1590/0100-6991e-20202722

**Published:** 2021-04-24

**Authors:** MÁRCIO CARDOSO KRAMBEK, JOÃO LUIZ VITORINO-ARAÚJO, RENAN MAXIMILIAN LOVATO, JOSÉ CARLOS ESTEVES VEIGA

**Affiliations:** 1 - Irmandade da Santa Casa de Misericórdia de São Paulo, Anestesiologia - ISCMSP, SP, Brasil; 2 - Irmandade da Santa Casa de Misericórdia de São Paulo, Neurocirurgia - ISCMSP, SP, Brasil; 3 - Faculdade de Ciências Médicas da Santa Casa de São Paulo (FCMSCSP), São Paulo, SP, Brasil; 4 - Hospital Sírio Libanês - São Paulo, SP, Brasil; 5 - Hospital HCOR - São Paulo, SP, Brasil

**Keywords:** Anesthesia, Craniotomy, Scalp, Glioma, Neurosurgery, Anestesia, Craniotomia, Couro Cabeludo, Glioma, Neurocirurgia

## Abstract

The anesthesia for awake craniotomy (AC) is a consecrated anesthetic technique that has been perfected over the years. Initially used to map epileptic foci, it later became the standard technique for the removal of glial neoplasms in eloquent brain areas. We present an AC anesthesia technique consisting of three primordial times, called awake-asleep-awake, and their respective particularities, as well as delve into the anesthetic medications used. Its use in patients with low and high-grade gliomas was favorable for the resection of tumors within the functional boundaries of patients, with shorter hospital stay and lower direct costs. The present study aims to systematize the technique based on the experience of the largest philanthropic hospital in Latin America and discusses the most relevant aspects that have consolidated this technique as the most appropriate in the surgery of gliomas in eloquent areas.

## RESUMO

A anestesia para craniotomia em paciente acordado (CPA ou awake craniotomy) é técnica anestésica consagrada e aperfeiçoada ao longo dos últimos anos. Utilizada inicialmente para mapeamento de focos epilépticos, consolidou-se posteriormente como técnica padrão para a remoção de neoplasias de origem glial em áreas eloquentes cerebrais. A técnica de anestesia CPA apresentada constitui-se em três tempos primordiais denominados acordado-dormindo-acordado (asleep-awake-asleep) e respectivas particularidades, assim como o manejo quanto às medicações anestésicas utilizadas de forma pormenorizada. A utilização em gliomas de baixo e de alto grau se demonstrou favorável para a ressecção de tumores dentro dos limites funcionais dos pacientes, com menor tempo de internação hospitalar e de custos diretos. O presente estudo visa realizar a sistematização da técnica baseada na experiência do maior Hospital Filantrópico da América Latina e discute os aspectos mais relevantes que consolidaram essa técnica como a mais adequada na cirurgia dos gliomas em áreas eloquentes.

## INTRODUCTION

The anesthesia for awake craniotomy (AC) was first performed by Sir Victor Horsley, in 1886, to locate epileptic foci with the aid of cortical electrical stimulation^1^. Wilder Penfield, a neurosurgeon and researcher, made mappings in conscious patients with severe epilepsy under local anesthesia, directly observing the brain and assessing responses to electrical stimuli. He prepared detailed reports on anatomical and functional brain correlation^2^. Since the 1980s, some authors have consolidated this technique in the treatment of neoplasms of glial origin. AC is usually carried out in the resection of tumors in eloquent brain areas, allowing the intraoperative functional mapping and identification of the regions related to language, motility, sensitivity, and vision^3^ ^,^ ^4^. There is evidence that this strategy reduces the period of postoperative recovery, hospital stay, and costs^5^ ^,^ ^6^. The main objective of AC is to minimize the risk of neurological damage, while maximizing the resection of the tumor, allowing a more radical removal, with consequent increase in survival^7^ ^,^ ^8^.

Several studies have been published, but few completely and critically expose the technique and the anesthetic tactics adopted. Thus, the present work aims to revisit the main nuances and describe the systematization of the technique used in 20 patients, in the oncology neurosurgery sector of the largest philanthropic hospital in Latin America, the Santa Casa de Misericordia de Sao Paulo.

### Anesthetic techniques

Several anesthetic techniques and tactics are adopted for performing AC by different researchers and services. These can be a combination of two or more anesthetic agents, such as propofol, remifentanil, and dexmedetomidine. Local infiltration of the entry points of the Mayfield skull support fixation pins is done with local anesthetic. Some services use the scalp block technique^9^. The result of the anesthetic technique for the surgical procedure may vary according to the choice and directly affect the surgical result, as well as postoperative analgesia^9^ ^-^ ^11^.

### Anesthetic technique at the Centre Hospitalier Universitaire de Montpellier (CHU)

During a fellowship at the neuroanesthesia service of the CHU, a recognized worldwide center in the use of AC for resection of low grade gliomas, we observed a systematization of the anesthetic technique, composed of three phases: asleep, awake, and asleep. The first and third stages are performed with the patient under general anesthesia, with laryngeal mask ventilation and continuous infusion of remifentanil and propofol. Local anesthesia is performed at the insertion points of the Mayfield-type skull fixator pins. The second phase, when specific neuropsychomotor tests are carried out, is performed with the patient awake and continuously monitored^12^.

### Anesthetic technique for AC used at the Central Hospital of the Irmandade da Santa Casa de Misericórdia de São Paulo.

The anesthetic technique is based on the pillars adopted by the Montpellier school (asleep/awake/asleep), but with adaptations. These include a scalp block with 1% lidocaine without vasoconstrictor associated with 0.5% bupivacaine with epinephrine, in a 50-50% dilution, followed by an intraoperative blockade of the surgical flap (temporalis muscle block in two stages, with 0.75% ropivacaine), plus a dura mater anesthesia with gauze soaked with 1% lidocaine without vasoconstrictor, for 10 minutes. The scalp anesthesia is again performed after the patient’s awakening in the AC third stage in case he/she presents a pain visual analog scale greater than or equal to six.

## TECHNIQUE DESCRIPTION

### First stage - ASLEEP

We perform a venipuncture (18 or 20 Fr gauge catheter) in the limb of the same side to be evaluated by the neurophysiology staff, for example, for a right side tumor, the neurophysiological assessment of the left side, preferably the puncture is on the right limb, since the corticospinal and spinothalamic traits are crossed. Pre-oxygenation starts with mask, followed by injection of 1% lidocaine without epinephrine for pain prevention at the injection site. General anesthesia is induced with propofol and remifentanil, up to the expected target dose of 6-8 µg/mL of the former and 4 ng/mL of the latter. The laryngeal mask is introduced, and the patient is coupled to mechanical ventilation, with controlled pressure mode at a maximum peak pressure of 16 cm/H_2_O, without positive end-expiratory pressure (PEEP). After adapting the laryngeal mask, the target dose of medications should be titrated until reaching the bispectral index (BIS) between 40 and 60. Even at this stage, it is important to check the patient’s position with attention to the extremities, limbs, and eye occlusion after placing ophthalmic gel on the cornea. As soon as the patient is coupled to mechanical ventilation, a new peripheral venipuncture is performed with a 20 or 18 Fr catheter and an arterial line is punctured. Then, urinary catheterization and thermometer placement take place. There is no need to insert a central venous catheter. We administer prophylactic antibiotics (cefuroxime 1.5 g, which should be repeated every 4 hours after the procedure) and antiemetic medication associated with corticosteroids (8 mg ondansetron and 10 mg dexamethasone).

At this stage, we also perform the scalp block using a combination of two local anesthetics, 1% lidocaine without vasoconstrictor associated with 0.5% bupivacaine with epinephrine (15-20 mL of the solution). The bilaterally blocked nerves are the supraorbital, supratrochlear, auriculotemporal, greater occipital, and lower occipital. Additionally, we inject the fixing points of the Mayfield support and the surgical incision site with 2% lidocaine with epinephrine (10-15 mL). After the block, the patient can be properly positioned on the Mayfield skull support. 

### Second phase - AWAKE

This phase starts with the suspension of Propofol and remifentanil, after placement of gauze soaked in a 1% lidocaine without vasoconstrictor (10 mL) on the dura mater for 10 minutes. The eye cover is removed, excessive stimulation of the patient is avoided, and ambient noise is controlled. The ventilator is placed in the spontaneous ventilation mode, with the BIS above 60-70 and the patient resumes spontaneous ventilation. As soon as the appropriate oxygen saturation is obtained, the laryngeal mask can be removed. There is no need for oxygen supplementation during the awake phase. In this phase, some medications and measures can be adopted, aiming at the patient’s comfort. If he/she experiences tremors, an intravenous dose of clonidine, 20 to 30 mcg, can be administered. In case of nausea, 6 to 10 mg of dexamethasone. If the patient has seizures, the surgeon must bathe the exposed brain with cold saline (5-10 degrees Celsius). At this stage, it is extremely important that the patient is in verbal contact and undergoing neuro-linguistic tests (Figure 2), so that any agitation is avoided. It is at this stage that the patient will perform all cognitive and motor tests suggested by the neurophysiologist and/or neuropsychologist, while the neurosurgeon, through electrical stimulation (Figure 3) with a bipolar probe and the Penfield technique (Figure 4), performs the cortical mapping and demarcates the patient’s functional area.

**Figure 1:**
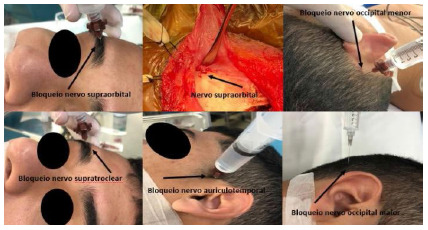
Scalp block for post-craniotomy analgesia (image use authorized by the patient).

**Figure 2:**
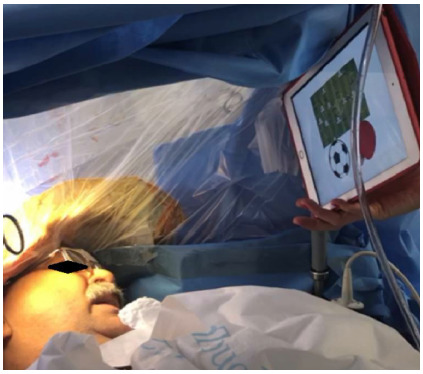
Patient undergoing tests specific to nomination, semantics, fluency, and verbal memory during resection of a brain tumor in the language area (image use authorized by the patient).

**Figure 3:**
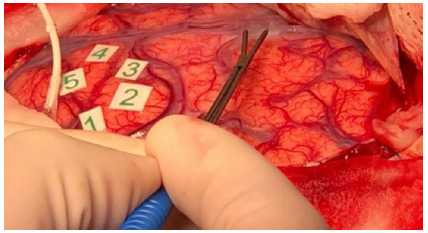
Cortical mapping with a bipolar probe using the Penfield technique (image use authorized by the patient).

**Figure 4:**
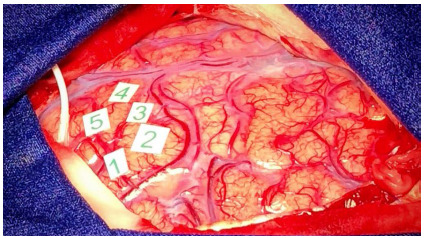
Demarcation of the language functional area. Stimulation of the region delimited by numbers interrupted speech articulation (image use authorized by the patient).

 Eventually, the patient may complain of pain related to position or even in the surgical field and if this happens, remifentanil can be infused in a low controlled dose (target TCI of 0.1 to 0.2 ng/mL). If the patient has a blood pressure cuff, it is necessary to space its measurement to every 10 minutes. Elevation in blood pressure levels is not necessarily a concern, as cerebral self-regulation is now established and the need to intervene occurs only if the patient displays measurements above 170 mm/Hg of systolic pressure. In this case, one can introduce titrated vasodilators, such as nitroglycerin. 

### Third phase - ASLEEP

In this third phase, after tumor resection, we perform a new general anesthesia. After pre-oxygenation, the patient receives induction with remifentanil and propofol again, and if there is no need for continuity of sensory or motor monitoring, muscle relaxants can be administered. In this case, there is need for intubation, with the aid of a videolaryngoscope. If muscle relaxant is not administered, the patient may have the laryngeal mask inserted again for the anesthesia sequence. In both cases, the patient is again placed under controlled mechanical ventilation. At this time, prednisolone (125 - 250 mg) can be administered, which must be maintained for 24 hours every 8 hours.

## DISCUSSION

Modern anesthesia requires that the anesthesiologist have a comprehensive medical knowledge to take vital decisions in a short time. The availability of equipment and parameters in the operating room aids in total control of anesthesia, be it inhaled, mixed, or entirely venous. The use of total intravenous anesthesia (TIVA) promotes better maintenance of brain conditions, being fundamental to tumor removal, which must be as radical as possible^13^. TIVA has neuroprotective properties, reduces the intracranial pressure (ICP), cerebral blood flow (CBF), cerebral metabolic rate (CMR), and edema, as well as allowing the rapid induction and recovery, being the most used in cases of brain neoplasms^14^ ^,^ ^15^. Keeping the brain in appropriate conditions facilitates dynamic manipulation of structures, reduces intraoperative edema, and the risks in the postoperative period. This outcome was observed in a meta-analysis involving 1,819 patients undergoing elective craniotomy under TIVA, wherein in addition to increased hemodynamic stability during surgery and CBF, there were lower incidences of nausea and vomiting in the postoperative period related to cerebral edema and increased ICP. Additionally, there were smoother awakening and less cough when compared with inhalation anesthesia^16^. The anesthesiologist must be skilled in reestablishing a conscious level suitable for performing the specific neuropsychological tests, this being possible only by meticulous balancing of sedation and analgesia^17^.

The anesthetic technique used in AC varies according to the experience of professionals and the availability of resources. ACs are based on protocols with different anesthetic medications and intra and postoperative analgesia, either by adopting the scalp block^18^ or by infiltration of the incision with of local anesthetics. The expected outcome is based on patient comfort during the operation and on the rapid recovery of cognitive ability for neurological assessment. AC methods with the patient awake throughout the whole procedure prioritize carrying out the scalp block as a comfort and analgesia method, while techniques that use an asleep phase prioritize infiltration only at the incision site, with or without postoperative opioids. In the awake-awake-awake method, also called awake throughout approach, the patient remains awake during the entire surgical time, with intraoperative analgesia promoted only by the scalp block. In this case, the researchers avoid the use of hypnotics and fast-acting opioids, such as propofol and remifentanil, fearing the occurrence of intraoperative respiratory depression. Although less used, Shafiq et al. reported a lower rate of intraoperative complications with this technique^19^. Regarding the scalp block, it is important to report that this technique is not used by all anesthesiologists. In some countries, the health system is financed by social insurance and any additional craniotomy procedures are associated with higher costs for the system. In addition, experience in performing the scalp lock is particularly important for the procedure, as it can directly interfere in intra and postoperative results^20^.

Stevanovic et al., in a systematic review, compared methods in which the patient remains awake throughout the operation and another in which the patient wakes up only for cognitive assessment (asleep-awake-asleep)^9^. In cases where the controlled target infusion technique (CTIT), or asleep-awake-asleep, was used, the authors noticed that the adoption of dexmedetomidine allowed a reduction in the consumption of opioids and propofol, as well as maintaining better hemodynamic stability. There was also a lower incidence of respiratory depression, despite a higher incidence of bradycardia. The benefit of both techniques consists in reducing the consumption of sedative medications during the operation, thus improving the capacity for neurological mapping^21^ ^,^ ^22^. Studies conducted by Özlü and Chowdhury et al. demonstrated less interference in the electroencephalogram by dexmedetomidine when compared with propofol in AC procedures. However, the association of remifentanil and propofol allows a much faster cognitive recovery after awakening than dexmedetomidine in the evaluation of cognitive tests^23^ ^,^ ^24^.

The occurrence of focal epileptic seizures during brain manipulation is one of the adverse events that requires rapid intervention. There is no need for prophylactic prevention, as the crisis can occur in the manipulation or traction of a certain brain structure. However, if it occurs intraoperatively, cold saline can be instilled over the exposed brain, without the need for intravenous medication. On the other hand, if the seizure is generalized, an antiepileptic agent should be promptly administered, bearing in mind that these agents can cause sedation and respiratory depression, rendering a probable negative outcome in AC^24^.

Analgesia in AC is performed through the infiltration of the scalp under the surgical incision line and through the scalp block performed bilaterally at the previously mentioned points^25^, as well as by the infiltration of the muscle flap. The use of conventional analgesics is not remarkably effective in controlling pain and the use of opioids is associated with impairment in the performance of specific neuropsychological tests, due to the drowsiness and cognitive impairment they produce. The neurosurgeon must be meticulous, preferably using transcortical access, avoiding traction of vascular structures and the use of bipolar coagulation near the dura at the cranial base^26^.

Gliomas, whether of low or high degree of malignancy, when present in eloquent areas of the brain, remain a challenge for neurosurgeons and anesthesiologists. These tumors used to be resected under general anesthesia, but the advent of AC changed this reality, allowing an oncofunctional balance, that is, more radical surgical removal associated with the preservation of neurological functions. Until then, only low-grade gliomas were resected by the AC technique with satisfactory outcomes, but new studies have found evidence of the applicability of the technique also for high-grade gliomas, with good neurological outcome when compared with general anesthesia^12^.

Although several works have verified the quality of the AC technique, further studies with protocols and more patients are needed to prove the superiority of one technique over the other. In addition, it is a technique that has little demand for equipment and materials and promotes a significant reduction in direct hospital costs. In view of this, the systematization of the AC technique becomes relevant and applicable in countries with scarce health resources, such as Brazil.

## CONCLUSION

Anesthesia for awake craniotomy (AC) is a strategy that has been enhanced over time by adding modern techniques of cortical mapping, development of drugs, and methods of continuous infusion. It allows the patient’s neurological functions to be continuously evaluated, promoting an increase in the degree of brain tumor resection, associated with functional neurological preservation. The adoption of scalp block offers comfort to the patient, in addition to reducing the consumption of anesthetic medications in the intraoperative period, as well as strict pain control in the postoperative one. Associated with the absence of invasive procedures (non-invasive airway device, central venous puncture, and peripheral arterial puncture), the procedure allows for shorter hospital stays and early return to daily activities. The refinement of this anesthetic technique seeks to improve the prognosis of neuro-oncological patients and to allow other professionals to replicate it in their institutions.
